# Clinical characteristics and antimicrobial therapy of healthcare-associated carbapenem-non-susceptible gram-negative bacterial meningitis: a 16-year retrospective cohort study

**DOI:** 10.1186/s12879-024-09237-9

**Published:** 2024-04-02

**Authors:** Jiyan Xu, Xiaoling Du, Dan Li, Pei Li, Qinglan Guo, Xiaogang Xu, Fupin Hu, Minggui Wang

**Affiliations:** 1 Institute of Antibiotics, Huashan Hospital, Fudan University, Shanghai, 200040 China; 2 Key Laboratory of Clinical Pharmacology of Antibiotics, National Heath Commission of People’s Republic of China, Shanghai, China

**Keywords:** Meningitis, Gram-negative bacteria, Carbapenem-resistance, Risk factor, Antimicrobial therapy

## Abstract

**Objective:**

Healthcare-associated Gram-negative bacterial meningitis is a substantial clinical issue with poor outcomes, especially for neurosurgical patients. Here, we aimed to study the characteristics and treatment options of patients with healthcare-associated carbapenem-non-susceptible (Carba-NS) Gram-negative bacterial meningitis.

**Methods:**

This observational cohort study was conducted at a teaching hospital from 2004 to 2019. The clinical characteristics of patients with meningitis with Carba-NS and carbapenem-susceptible (Carba-S) bacilli were compared, and the antimicrobial chemotherapy regimens and outcomes for Carba-NS Gram-negative bacterial meningitis were analyzed.

**Results:**

A total of 505 patients were included, of whom 83.8% were post-neurosurgical patients. The most common isolates were *Acinetobacter* spp. and *Klebsiella* spp., which had meropenem-resistance rates of 50.6% and 42.5%, respectively, and showed a markedly growing carbapenem-resistance trend. Kaplan–Meier curve analysis revealed that Carba-NS Gram-negative bacilli were associated with a significantly higher in-hospital mortality rate (18.8%, 35/186) compared to the Carba-S group (7.4%, 9/122; *P* = 0.001). For Carba-NS *Enterobacterales* meningitis, aminoglycoside-based and trimethoprim-sulfamethoxazole-based regimens yielded significantly higher clinical efficacy rates than non-aminoglycoside-based and non-trimethoprim-sulfamethoxazole-based regimens (69.0% vs. 38.7%, *P* = 0.019 and 81.8% vs. 46.9%, *P* = 0.036, respectively). For Carba-NS *A. baumannii* complex meningitis, tetracycline-based (including doxycycline, minocycline, or tigecycline) therapy achieved a significantly higher clinical efficacy rate (62.9%, 22/35) than the non-tetracycline-based therapy group (40.4%, 19/47; *P* = 0.044).

**Conclusions:**

Our findings revealed that Carba-NS Gram-negative bacilli are associated with higher in-hospital mortality in patients with healthcare-associated meningitis. The combination therapies involving particular old antibiotics may improve patients’ outcome.

**Trial registration:**

This study was registered on the Chinese Clinical Trial Register under ChiCTR2000036572 (08/2020).

**Supplementary Information:**

The online version contains supplementary material available at 10.1186/s12879-024-09237-9.

## Background

Meningitis poses a great challenge to human health, with poor outcomes and high hospital costs [1]. Due to vaccination programs, community-acquired meningitis presents a decreasing trend [2]. However, with the development of neurosurgery, healthcare-associated central nervous system (CNS) infection has become a substantial clinical issue, with an incidence ranging from 0.3 to 7.4% and a high mortality rate ranging from 8 to 34%, as reported previously [3, 4]. Gram-negative bacilli, especially *Acinetobacter baumannii* and *Klebsiella pneumoniae*, have emerged as predominant pathogens [5].

Currently, carbapenem-resistant Gram-negative bacilli represent a considerable clinical threat [6, 7]. According to the China Antimicrobial Resistance Surveillance Network (CHINET) program, the resistance rates of *A. baumannii* and *K. pneumoniae* isolated from the cerebrospinal fluid (CSF) to meropenem show an increasing trend with rates of 85.6% and 64.1% in 2018, respectively, both of which were much higher than those isolated from other specimens (blood, urine, and lower respiratory tract) [8]. Available active antimicrobial agents are limited to those for carbapenem-resistant Gram-negative bacilli. For carbapenem-resistant Enterobacterales (CRE) infection, active agents include tigecycline, polymyxins, aminoglycosides, fosfomycin, and their combination with carbapenems or trimethoprim-sulfamethoxazole (SXT) [9, 10]. For carbapenem-resistant *A. baumannii* (CRAB) infection, options included tigecycline, polymyxins, sulbactam (either in its standalone form or as part of a fixed-dose combination), aminoglycosides, fosfomycin, and their combination with minocycline, doxycycline, or carbapenems [9]. Several possible treatment options for CRE are still unavailable in China, such as meropenem-vaborbactam, imipenem-cilastin-relebactam, cefiderocol, and plazomicin [11], while polymyxins and ceftazidime-avibactam remained unavailable until 2018 and 2019, respectively. Considering the poor penetration rate of most antibiotics through the blood-CSF and blood-brain barrier, treatment of meningitis, especially multidrug-resistant bacterial meningitis, is challenging. Thus, combination therapy is widely adopted to improve the outcome of meningitis patients [12, 13]. Nevertheless, further analysis of combinations with particular agents is expected to show which solution is associated with clinical success.

The Huashan Hospital affiliated to Fudan University is a tertiary hospital with more than 2,000 beds, while the neurosurgery department is one of the largest centers in Asia and the Pacific, with more than 600 beds and more than 10,000 annual neurosurgeries.

In this study, we reviewed the clinical data of adult inpatients with Gram-negative bacterial meningitis admitted to the Huashan Hospital from 2004 to 2019 and then compared carbapenem-non-susceptible (Carba-NS) and carbapenem-susceptible (Carba-S) Gram-negative bacterial meningitis cases. In the current study, we aimed to identify the distribution and antibiotic resistance of pathogens and the clinical characteristics and risk factors of Gram-negative bacterial meningitis. For Carba-NS Enterobacterales and *A. baumannii* complex meningitis cases, we assessed the efficacy of different antimicrobial therapy regimens and compared the effects and outcomes of different therapies.

## Methods

### Settings and study design

During the 16-year study period from January 1, 2004 to December 31, 2019, patients with one or more positive Gram-negative CSF cultures were reviewed. Only the first culture sample was selected for a patient in one continuous admission. During the study period, routine clinical practices were maintained consistently in the hospital, while the infection prevention and control measures became progressively better with time.

The diagnosis of healthcare-associated meningitis must meet the criteria established by the Center for Disease Control and Prevention and National Healthcare Safety Network (CDC/NHSN) [14]. Patients who had positive culture without signs, symptoms, and alteration of CSF were considered to have polluted or colonized samples and were excluded from the study. Clinical data of Gram-negative healthcare-associated meningitis were abstracted and recorded, including demographic information, reasons for admission, comorbidity, invasive operations and surgeries performed before isolation, laboratory results, antimicrobial therapies and responses, and clinical outcomes. The patients with meningitis were followed-up with until discharge or death. Cases with high volumes of lost data were excluded from the study.

### Variables and definitions

In this study, isolates that were susceptible to both imipenem and meropenem by current Clinical and Laboratory Standards Institute (CLSI) breakpoints were categorized as Carba-S, while the remaining strains were considered as Carba-NS. In particular, the minimal inhibitory concentrations (MICs) of strains including *Proteus* spp., *Providencia* spp., and *Morganella morganii* to imipenem tend to be higher; thus, such isolates must be resistant or intermediate to meropenem to meet the Carba-NS definition [15].

The Glasgow Coma Scale (GCS) and the Glasgow Outcome Scale (GOS) [16] were used to evaluate the consciousness and outcome of patients on admission and discharge, respectively. Glucocorticoid use was defined as the receipt of ≥ 10 mg/d of prednisone for 5 days in the past month. The antimicrobial therapies of each patient were defined as agents against Gram-negative bacteria applied after the antimicrobial susceptibility results were available and continued for at least 3 days. The response to therapy was evaluated at the end of the treatment. Clinical success was defined as the resolution of signs and symptoms of meningitis (e.g., fever, headache, nausea, vomiting, lethargy, meningeal irritation, and cranial nerve signs), ease of CSF parameters (e.g., cell count, glucose, and protein testing), and sterilization of CSF cultures (three consecutive CSF cultures from separate days produced negative results) [17]. Patients who did not meet any of these criteria were classified as clinical failure. For fatal cases, deaths related to meningitis were categorized as clinical failure. However, cases in which meningitis showed significant improvement, meeting the aforementioned criteria for clinical success, but later resulted in death due to other causes, were still categorized as clinical success.

### Bacterial identification and antimicrobial susceptibility testing

All of the non-duplicated Gram-negative isolates from healthcare-associated meningitis patients were collected and stored at − 80 °C. Resuscitated strains were identified by 16 S rRNA gene sequencing, and antimicrobial susceptibility testing was performed using the broth dilution method. The results were interpreted based on CLSI document M100-S31. Besides, the MICs of cefoperazone-sulbactam were determined according to the recommendation by R. N. Jones et al. [18], with MICs ≤ 16/8, 32/16, and ≥ 64/32 µg/ml interpreted as susceptible, intermediate, and resistant, respectively. The MICs of tigecycline were interpreted according to the recommendation of the Food and Drug Administration, with MICs ≤ 2, 4, and ≥ 8 µg/ml categorized as susceptible, intermediate, and resistant, respectively.

**Fig. 1:**
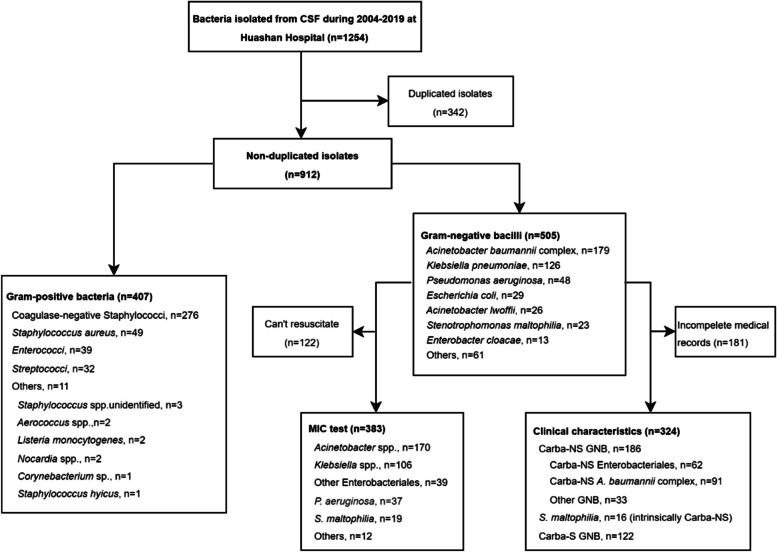
Flowchart of inclusion criteria. Carba-NS: Carbapenem-non-susceptible, GNB: Gram-negative bacilli, Carba-S: Carbapenem-susceptible

The flowchart for study inclusion is shown in Fig. 1.

### Statistical analysis

Categorical variables are described as percentages and were evaluated using the Chi-squared test or Fisher’s exact test, as appropriate. Normally and non-normally distributed continuous variables were compared using the independent-samples *t-*test and the Mann–Whitney *U* test, respectively. Binary logistic regression was performed to analyze multiple risks of Carba-NS Gram-negative bacterial meningitis and factors relating to clinical success. Survival curves were prepared using the Kaplan–Meier method, with the log-rank test applied to compare survival distributions. Cox regression analysis was performed to identify risk factors for in-hospital mortality of patients with meningitis. All *P*-values were two tailed, and statistical significance was set at *P* < 0.05. Statistical analyses were performed using GraphPad Prism 9 (GraphPad Prism Software Inc., San Diego, CA, USA) and IBM SPSS Statistics for Windows v.20.0 (IBM Corp., Armonk, NY, USA).

## Results

### Bacterial isolates and antimicrobial susceptibility

From January 1, 2004 to December 31, 2019, 912 non-duplicate CSF isolates were collected from adult inpatients. Of these, 407 (44.6%) were Gram-positive cocci and 505 (55.4%) were Gram-negative bacilli. Among the Gram-negative bacilli isolates, 423 (83.8%) were collected from post-surgical patients.

**Fig. 2:**
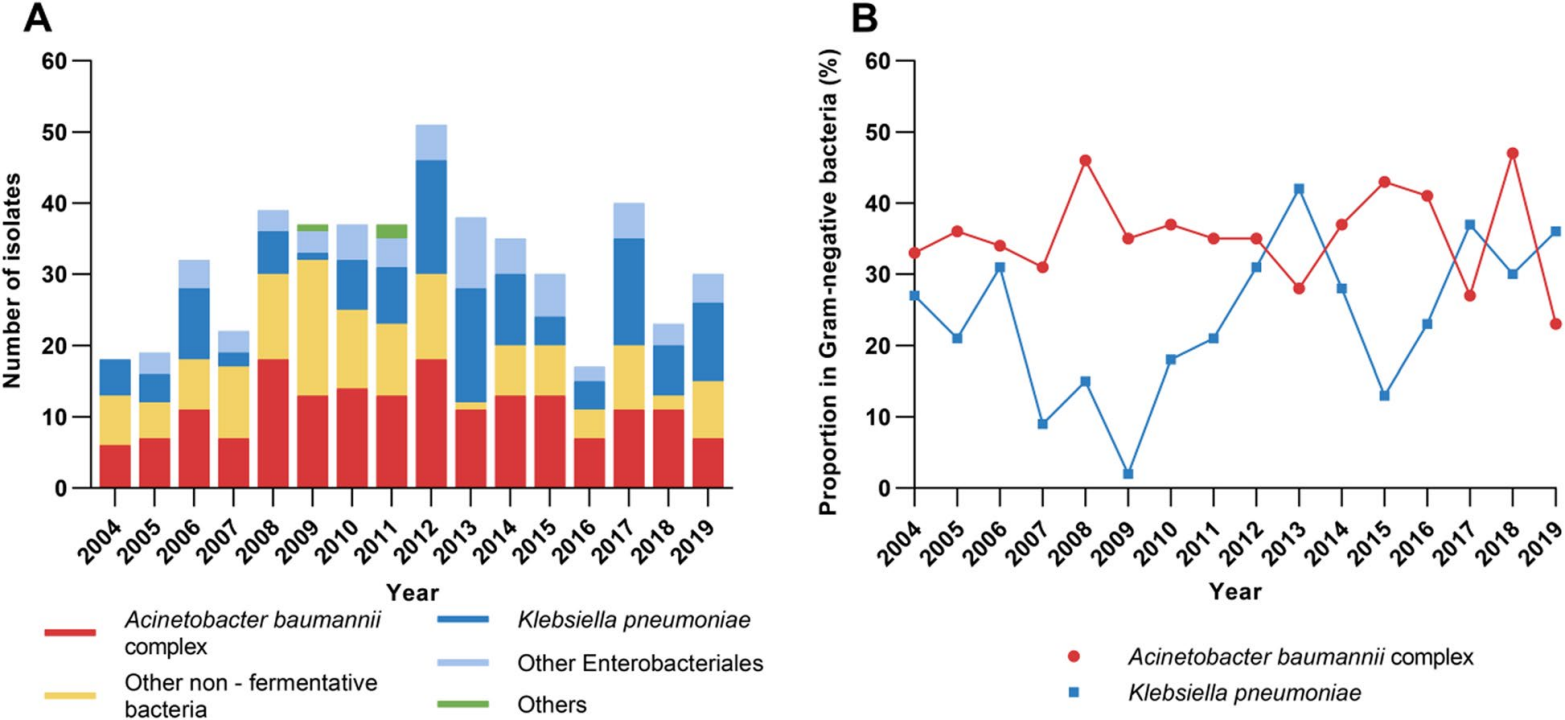
Distribution of Gram-negative bacteria isolated from the central nervous fluid of adult inpatients with healthcare-associated meningitis from 2004 to 2019 (*n* = 505). **A **Proportion of *Acinetobacter baumannii* complex, other non-fermentative bacteria, *Klebsiella pneumoniae*, other Enterobacterales, and other species isolated from the CSF per year. **B **Trend of the proportion of *Acinetobacter baumannii* complex and *Klebsiella pneumoniae* isolated from the CSF per year

The predominant seven groups of bacilli accounted for 87.9% (444/505) of Gram-negative isolates: *A. baumannii* complex (35.4%, 179/505), *K. pneumoniae* (25.0%, 126/505), *Pseudomonas aeruginosa* (9.5%, 48/505), *Escherichia coli* (5.7%, 29/505), *A. lwoffii* (5.1%, 26/505), *Stenotrophomonas maltophilia* (4.6%, 23/505), and *Enterobacter cloacae* (2.6%, 13/505). Other Enterobacterales included *K. aerogenes* (*n* = 6), *Proteus mirabilis* (*n* = 5), *K. oxytoca* (*n* = 4), *Serratia marcescens* (*n* = 4), and others (*n* = 4). Other non-fermentative bacteria included other *Pseudomonas* species (*n* = 5), *Chryseobacterium indologenes* (*n* = 6), *A. junii* (*n* = 5), *Comamonas testosteroni* (*n* = 4), *Elizabethkingia meningoseptica* (*n* = 3), *Brevundimonas diminuta* (*n* = 3), and others (*n* = 7). During the 16-year period, *A. baumannii* complex remained the most common isolate, while the number of *K. pneumoniae* isolates showed an increasing trend (Fig. 2).

**Fig. 3:**
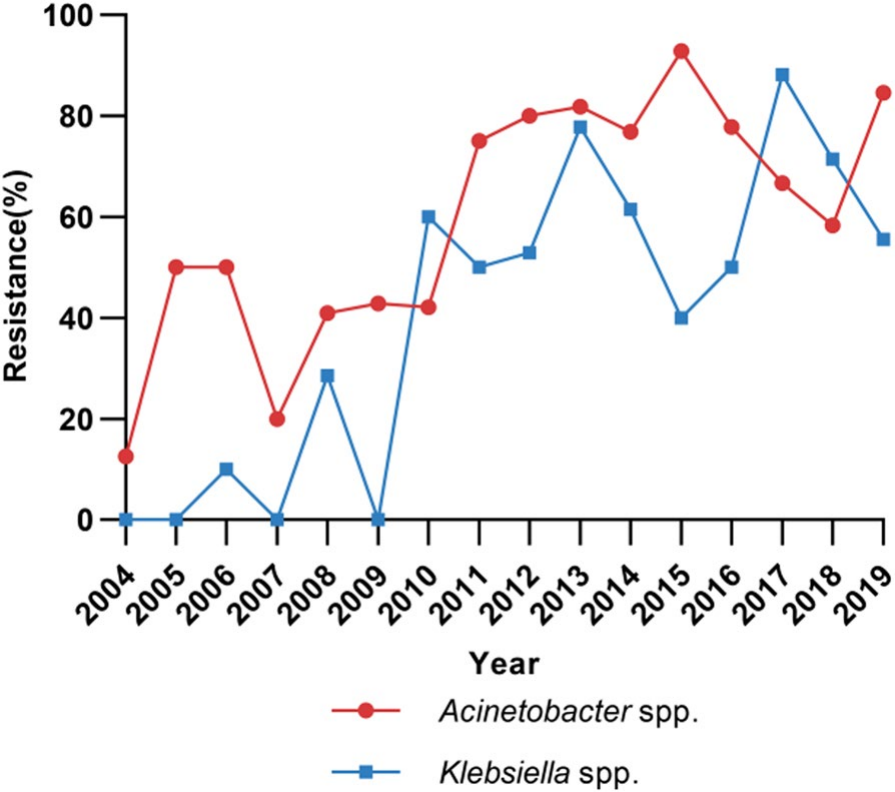
Trends of meropenem-resistance rates in *Acinetobacter* spp. (*n* = 170) and *Klebsiella* spp. (*n* = 106) isolated from cerebrospinal fluid

Of the 505 Gram-negative isolates, 122 strains did not resuscitate; thus, antimicrobial susceptibility testing with the broth dilution method was performed in 383 strains (Table 1). The resistance rates of *Acinetobacter* spp. (*n* = 170) to imipenem and meropenem were as high as 50.0% and 50.6%, respectively. For *Klebsiella* spp. (*n* = 106), the resistance rates to imipenem and meropenem were 43.4% and 42.5%, respectively. During 2004 and 2013, a markedly increasing trend of the resistance to carbapenems was observed in both *Acinetobacter* spp. and *Klebsiella* spp., while the resistance rate fluctuated around 58.3–92.9% and 40.0–88.2% during 2014 and 2019, respectively (Fig. 3).

**Table 1:** Antimicrobial susceptibility of Gram-negative bacteria isolated from patients with healthcare-associated meningitis from 2004 to 2019 (*n* = 383)

	*Klebsiella* spp.^a^		Other Enterobacterales^b^		*Acinetobacter* spp.^c^		*Pseudomonas aeruginosa*		*Stenotrophomonas maltophilia*		Others^d^	
Antimicrobials	(*n* = 106)		(*n* = 39)		(*n* = 170)		(*n* = 37)		(*n* = 19)		(*n* = 12)	
	S (%)	R (%)	S (%)	R (%)	S (%)	R (%)	S (%)	R (%)	S (%)	R (%)	S (%)	R (%)
Piperacillin	7.5	90.6	23.1	76.9	11.2	75.9	54.1	13.5	NA	NA	58.3	33.3
Piperacillin-tazobactam	40.6	58.5	84.6	15.4	22.4	65.9	62.2	8.1	NA	NA	58.3	41.7
Cefotaxime	13.2	86.8	25.6	74.4	10.6	77.6	NA	NA	NA	NA	8.3	50.0
Ceftazidime	20.8	73.6	51.3	48.7	21.2	78.2	62.2	37.8	31.6	52.6	16.7	75.0
Cefepime	35.8	55.7	46.2	35.9	24.1	57.1	73.0	13.5	NA	NA	8.3	83.3
Cefoperazone-sulbactam	28.3	55.7	64.1	23.1	35.3	22.9	62.2	16.2	73.7	21.1	58.3	33.3
Imipenem	50.9	43.4	94.9	5.1	40.6	50.0	48.6	24.3	NA	NA	33.3	66.7
Meropenem	51.9	42.5	97.4	2.6	42.9	50.6	37.8	27.0	NA	NA	16.7	66.7
Amikacin	49.1	50.9	76.9	23.1	26.5	72.9	78.4	21.6	NA	NA	16.7	66.7
Minocycline	59.4	24.5	48.7	33.3	48.8	25.3	NA	NA	84.2	5.3	100.0	0.0
Ciprofloxacin	28.3	67.0	43.6	56.4	15.9	81.8	62.2	37.8	NA	NA	8.3	75.0
SXT	56.6	43.4	46.2	53.8	37.1	62.9	NA	NA	73.7	26.3	75.0	25.0
Fosfomycin	67.0	31.1	76.9	23.1	NA	NA	NA	NA	NA	NA	NA	NA
Tigecycline	95.3	2.8	84.6	15.4	52.4	22.4	NA	NA	68.4	21.1	41.7	41.7

*Abbreviations*: *S *Susceptible, *R *Resistant, *NA *Not applicable, *SXT *Trimethoprim-sulfamethoxazole

^a^ *Klebsiella* spp.: *Klebsiella pneumoniae* (*n* = 98), *K. oxytoca* (*n* = 4), and *K. aerogenes* (*n* = 4)

^b^Other Enterobacterales: *Escherichia coli* (*n* = 19), *Enterobacter cloacae* (*n* = 13), *Serratia marcescens* (*n* = 3), *Proteus mirabilis* (*n* = 2), *Citrobacter freundii* (*n* = 1), and *Pantoea agglomerans* (*n* = 1)

^c^ *Acinetobacter* spp.: *Acinetobacter baumannii* complex (*n* = 147), *A. lwoffii* (*n* = 21), *and A. junii* (*n* = 2)

^d^Others: *Chryseobacterium indologenes* (*n* = 4), *Elizabethkingia meningoseptica* (*n* = 3), *Brevundimonas diminuta* (*n* = 2), *Alcaligenes xylosoxidans* (*n* = 1), *Pseudomonas putida* (*n* = 1), and *P. stutzeri* (*n* = 1)

### Clinical characteristics and risk factors of Carba-NS Gram-negative bacterial meningitis

Since the hospital information system only started reserving electronic data in 2016, the archives of some patients hospitalized before 2016 had information missing (*n* = 181). Therefore, a total of 324 cases were included for analysis of clinical characteristics. All of the 324 isolates collected from these patients had antimicrobial susceptibility testing reports from the Clinical Microbiology Department of the hospital, of which 299 were resuscitated and had MIC testing. The distribution of pathogens in these cases was as follows: (1) Enterobacterales (*n* = 124): *K. pneumoniae* (*n* = 89), *E. coli* (*n* = 15), *E. cloacae* (*n* = 4), *K. aerogenes* (*n* = 4), *S. marcescens* (*n* = 4), *P. mirabilis* (*n* = 3), and others (*n* = 5); (2) *A. baumannii* complex (*n* = 124); and (3) other Gram-negative bacteria (*n* = 76): *P. aeruginosa* (*n* = 27), *S. maltophilia* (*n* = 16), *A. lwoffii* (*n* = 13), *C. indologenes* (*n* = 4), *A. junii* (*n* = 4), *E. meningoseptica* (*n* = 3), and others (*n* = 9).

The clinical characteristics of the 308 patients with healthcare-associated Gram-negative bacterial meningitis (excluding 16 cases caused by *S. maltophilia*, which are intrinsically resistant to carbapenems) and comparison between the Carba-NS Gram-negative bacterial meningitis group (*n* = 186) vs. the Carba-S Gram-negative bacterial meningitis group (*n* = 122) are presented in Table 2. Most patients in both groups had an intensive care unit (ICU) stay, received neurosurgical procedures and general anesthesia, presented with fever, and showed increased white blood cell (WBC) and neutrophil counts in blood. Both groups of patients had increased levels of WBCs and multinuclear cells and decreased chloride and glucose in the CSF.

**Table 2:** Comparison of the clinical characteristics of meningitis caused by carbapenem-non-susceptible vs. carbapenem-susceptible Gram-negative bacteria

	Carba-NS group	Carba-S group		Univariate analysis		Multivariate analysis	
	(*n* = 186)	(*n* = 122)	*P*	OR (95% CI)	*P*	OR (95% CI)	*P*
Demographic information							
Male sex^a^, n (%)	136 (73.1)	72 (59.0)	**0.010**	1.889 (1.163–3.068)	**0.010**		
Age^b^, median (IQR)	50.0 (39.0–60.0)	44.5 (30.0–58.0)	**0.020**	1.018 (1.003–1.033)	**0.021**		
ICU stay^a^, n (%)	147 (79.0)	88 (72.1)	0.164				
GCS score^c^, n (%)					** < 0.001**		**0.001**
Severe (GCS 3–8)	118 (63.4)	41 (33.6)	** < 0.001**	4.053 (2.433–6.750)	** < 0.001**	2.935 (1.675–5.143)	** < 0.001**
Moderate (GCS 9–12)	19 (10.2)	12 (9.8)		2.230 (0.992–5.013)	0.052	1.743 (0.731–4.152)	0.210
Mild (GCS 13–15)	49 (26.3)	69 (56.6)					
Cerebrovascular disease, n (%)	72 (38.7)	33 (27.0)	**0.035**	1.703 (1.037–2.799)	**0.036**		
Traumatic brain injury, n (%)	70 (37.6)	26 (21.3)	**0.002**	2.228 (1.318–3.767)	**0.003**		
Intracranial tumor^a^, n (%)	31 (16.7)	52 (42.6)	** < 0.001**	0.269 (0.159–0.456)	** < 0.001**		
Comorbidities							
Diabetes^a^, n (%)	18 (9.7)	10 (8.2)	0.658				
Hypertension^a^, n (%)	48 (25.8)	23 (18.9)	0.156				
With non-CNS infection on admission^a^, n (%)	69 (37.1)	20 (16.4)	** < 0.001**	3.008 (1.711–5.287)	** < 0.001**	2.690 (1.459–4.960)	**0.002**
Hospitalization in the previous month before admission^a^, n (%)	125 (67.2)	61 (50.0)	**0.003**	2.049 (1.282–3.274)	**0.003**		
Predisposing factors							
Intra-cranial pressure monitor, n (%)	44 (23.7)	12 (9.8)	**0.002**	2.840 (1.432–5.635)	**0.003**	2.237 (1.052–4.755)	**0.036**
Ommaya^a^, n (%)	95 (51.1)	46 (37.7)	**0.021**	1.725 (1.083–2.747)	**0.022**		
Mechanical ventilation^a^, n (%)	98 (52.7)	41 (33.6)	**0.001**	2.200 (1.371–3.532)	**0.001**		
Carbapenem exposure^a^, n (%)	82 (44.1)	37 (30.3)	0.015	1.811 (1.118–2.935)	**0.016**	2.019 (1.195–3.413)	**0.009**
Deep vein intubation^a^, n (%)	100 (53.8)	52 (42.6)	0.056				
External ventricular drainage, n (%)	100 (53.8)	59 (48.4)	0.353				
General anesthesia^a^, n (%)	153 (82.3)	98 (80.3)	0.670				
Neurosurgical procedures^a^, n (%)	158 (84.9)	100 (82.0)	0.488				
Ventriculoperitoneal shunt, n (%)	22 (11.8)	29 (23.8)	**0.006**	0.430 (0.234–0.792)	**0.007**		
Continuous lumbar drainage^a^, n (%)	97 (52.2)	80 (65.6)	**0.020**	0.572 (0.357–0.917)	**0.020**		
Glucocorticoids^a^, n (%)	72 (38.7)	68 (55.7)	**0.003**	0.502 (0.316–0.797)	**0.004**		
Proton pump inhibitor, n (%)	125 (67.2)	98 (80.3)	**0.012**	0.502 (0.292–0.862)	**0.013**		
Treatment information							
Intrathecal therapy^a,d^, n (%)	15 (8.5)	6 (5.3)	0.301				
Length of the main antimicrobial therapy (days)^b,d^, mean ± SD	14.2 ± 1.0	19.5 ± 2.9	**0.048**				
Complications							
Hydrocephalus^a^, n (%)	78 (41.9)	62 (50.8)	0.126				
CSF leakage^a^, n (%)	25 (13.4)	11 (9.0)	0.237				
Surgical wound infection^a^, n (%)	29 (15.6)	16 (13.1)	0.547				
Fever^ad^, n (%)	174 (94.1)	109 (90.1)	0.198				
Organ failure^a,d^, n (%)	75 (41.2)	37 (31.6)	0.095				
Blood testing on the day of first positive culture							
WBC^c,d^, n (%)							
Decrease	5 (3.0)	1 (1.0)	0.995				
Normal range	57 (34.1)	38 (36.9)					
Increase	105 (62.9)	64 (62.1)					
Neutrophils^c,d^, n (%)							
Decrease	0 (0.0)	1 (1.0)	0.686				
Normal range	17 (10.2)	11 (10.7)					
Increase	150 (89.8)	91 (88.3)					
Platelet^c,d^, n (%)							
Decrease	19 (11.4)	4 (3.9)	0.496				
Normal range	101 (60.5)	81 (78.6)					
Increase	47 (28.1)	18 (17.5)					
Hypoalbuminemia^a,d^, n (%)	102 (62.6)	48 (48.0)	**0.020**				
Central nervous fluid testing							
WBC (× 10^6^/L)^b,e^, mean ± SD	4657.5 ± 788.5	3473.7 ± 980.0	0.345				
Multinuclear cell (%)^b,e^, mean ± SD	80.6 ± 1.5	76.0 ± 2.0	0.066				
Chloride (mmol/L)^b,e^, mean ± SD	114.0 ± 0.8	114.2 ± 0.9	0.882				
Glucose (mmol/L)^b,e^, mean ± SD	1.7 ± 0.1	2.0 ± 0.1	0.066				
Protein (mg/L)^b,e^, mean ± SD	5315.0 ± 377.0	4074.5 ± 511.3	**0.048**				
Co-infection with Gram–positive bacteria or MTB^a,d^, n (%)	41 (22.2)	35 (29.4)	0.154				
Outcome							
GOS score^c^, n (%)							
Good recovery	16 (8.6)	24 (19.7)	** < 0.001**				
Moderate disability	86 (46.2)	67 (54.9)					
Severe disability	5 (2.7)	3 (2.5)					
Vegetative state	44 (23.7)	19 (15.6)					
Dead	35 (18.8)	9 (7.4)					
Normalization of clinical symptoms^a^, n (%)	92 (49.5)	70 (57.4)	0.174				
Normalization of laboratory analysis in CSF^a,d^, n (%)	86 (51.5)	63 (63.0)	0.067				
CSF bacterial clearance rate^a^, n (%)	119 (64.0)	82 (67.2)	0.560				

*Abbreviations* *GCS* Glasgow Coma Scale, *GOS* Glasgow Outcome Scale, *MTB* Mycobacterium tuberculosis, *WBC* White blood cell

^a^Pearson’s Chi-square test

^b^Independent samples *t*-test

^c^Mann–Whitney *U* test

^d^Data partially missing

^e^Totally analyzed 289 cases (Carba-NS group, *n* = 175; Carba-S group, *n* = 114)

Compared to the Carba-S group, the Carba-NS group had a higher proportion of male patients (73.1% vs. 59.0%; *P* = 0.010), with older age (median age 50.0 vs. 44.5; *P* = 0.020), and lower GCS score (*P* < 0.001). The diagnosis was more frequently related to cerebrovascular disease (38.7% vs. 27.0%; *P* = 0.035) and traumatic brain injury (37.6% vs. 21.3%; *P* = 0.002) but was less common in intracranial tumors (16.7% vs. 42.6%; *P* < 0.001). Patients in the Carba-NS group also had more non-CNS infections, such as pneumonia, sepsis, and urinary tract infection on admission (37.1% vs. 16.4%; *P* < 0.001), as well as hospitalization in the previous month (67.2% vs. 50.5%; *P* = 0.003).

During hospitalization, the Carba-NS group patients more frequently received mechanical ventilation (52.7% vs. 33.6%; *P* = 0.001), intra-cranial pressure (ICP) monitor (23.7% vs. 9.8%; *P* = 0.002), Ommaya (51.1% vs. 37.7%; *P* = 0.021), with a higher rate of carbapenems exposure (44.1% vs. 30.3%; *P* = 0.015) but a lower proportion of administration of glucocorticoids (38.7% vs. 55.7%; *P* = 0.003), proton pump inhibitor (PPI) use (67.2% vs. 80.3%; *P* = 0.012), ventriculoperitoneal (VP) shunt (11.8% vs. 23.8%; *P* = 0.006), and continuous lumbar drainage (52.2% vs. 65.6%; *P* = 0.020), compared to the Carba-S group. On the first day of infection, patients in the Carba-NS group more frequently suffered from serum hypoalbuminemia (62.6% vs. 48.0%; *P* = 0.020), and the levels of protein in the CSF were higher (5315.0 ± 377.0 mg/L vs. 4074.5 ± 511.3 mg/L; *P* = 0.048) compared to those in the Carba-S group.

**Fig. 4:**
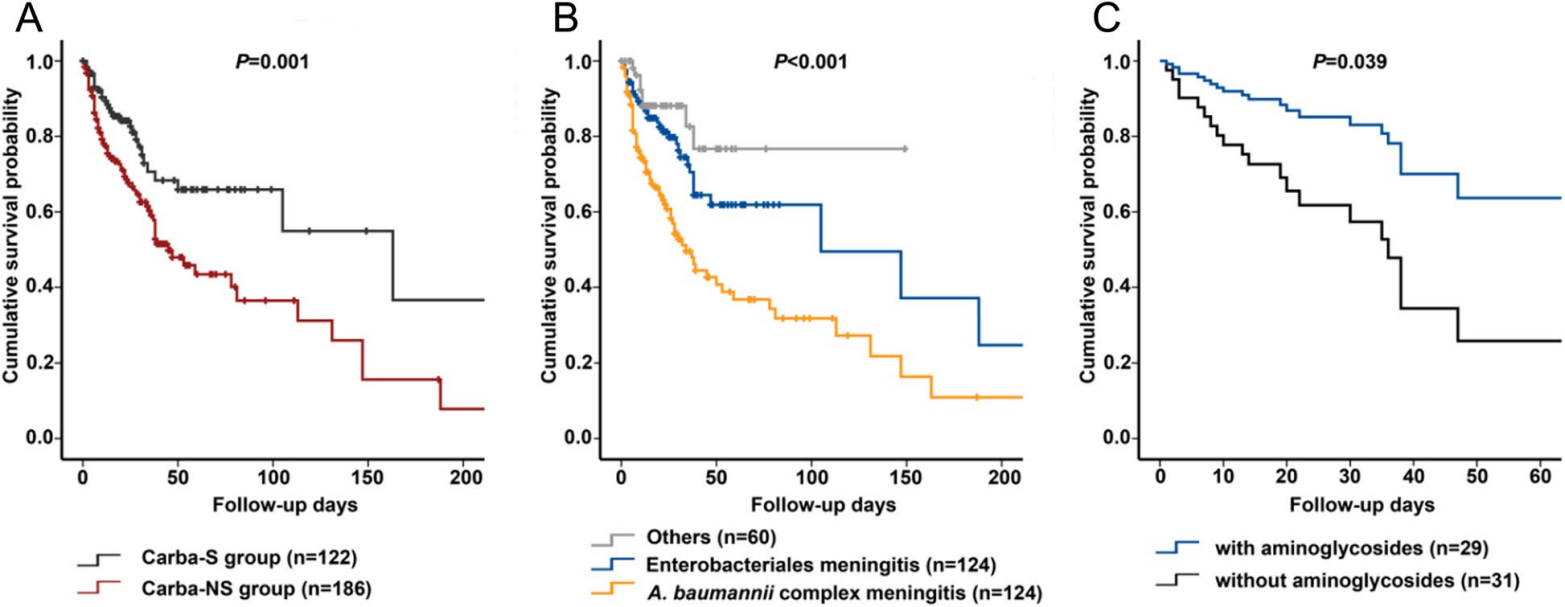
Survival analysis of patients with Gram-negative bacterial meningitis from 2004 to 2019. **A **Kaplan–Meier survival analysis of patients infected with Carba-NS Gram-negative bacterial meningitis compared to patients infected with Carba-S Gram-negative bacterial meningitis (*P* = 0.001). **B **Kaplan–Meier survival analysis of patients infected with *Acinetobacter baumannii* complex meningitis compared to patients infected with Enterobacterales meningitis and other Gram-negative bacterial meningitis (*P* < 0.001). **C **Cox survival analysis for the use of aminoglycosides for Carba-NS Enterobacterales meningitis (*P* = 0.039)

Carba-NS group patients have a higher in-hospital mortality rate than Carba-S group patients (18.8% vs. 7.4%; *P* = 0.005). Kaplan–Meier survival analysis also revealed that the rate of in-hospital mortality was significantly higher among patients infected with Carba-NS Gram-negative bacilli than those infected with Carba-S bacilli (*P* = 0.001). Moreover, the mortality rate among the *A. baumannii* meningitis group was significantly higher than that among the *K. pneumoniae* group and other Gram-negative bacterial groups (*P* < 0.001) (Fig. 4A and B).

Univariate analysis revealed that male sex, age, GCS score, cerebrovascular disease, traumatic brain injury, with non-CNS infection on admission, hospitalization in the month before admission, mechanical ventilation, carbapenems exposure, ICP monitor, and Ommaya were significantly associated with Carba-NS Gram-negative bacterial meningitis, whereas intracranial tumor, patients with glucocorticoids or PPI, VP shunt, and continuous lumbar drainage were associated with Carba-S Gram-negative bacterial meningitis. Multivariate analysis of these factors showed that independent risk factors associated with Carba-NS Gram-negative bacterial meningitis included severe coma (GCS 3–8) (odds ratio [OR]: 2.935; 95% CI: 1.675–5.143; *P* < 0.001), non-CNS infections on admission (OR: 2.690; 95% CI: 1.459–4.960; *P* = 0.002), application of ICP monitoring (OR: 2.237; 95% CI: 1.052–4.755; *P* = 0.036), and carbapenems exposure (OR: 2.019; 95% CI: 1.195–3.413; *P* = 0.009) (Table 2).

### Antimicrobial treatment and clinical outcomes of Carba-NS Gram-negative bacterial meningitis

Cases with detailed medical records of treatment and outcomes were collected. Except for *S. maltophilia* meningitis cases, the clinical success rate of the Carba-NS group (49.5%, 92/186) was lower than that of the Carba-S group (57.4%, 70/122), without statistical significance (*P* = 0.174). Enterobacterales and *A. baumannii* complex were two of the most common pathogens of Carba-NS meningitis.

### Carba-NS enterobacterales meningitis

Of the 124 cases of Enterobacterales meningitis, the clinical success rate of the Carba-NS group (51.6%, 32/62) was lower than that of the Carba-S group (67.7%, 42/62), with no statistical significance (*P* = 0.067). Among 62 patients with Carba-NS Enterobacterales meningitis, 60 (96.8%) patients received therapy involving active agents, with an efficacy rate of 53.3% (32/60), while two (3.2%) patients received therapy without active agents, and both cases failed to respond to treatment. The clinical efficacy rates of patients who received monotherapies and combination therapies were both 53.3% (8/15 vs. 24/45). Comparing the 2004–2009 group with the 2010–2019 group, the clinical efficacy rates (61.9% [13/21] vs. 59.2% [61/103]) showed no significant differences (*P* = 0.819).

For the 60 cases of Carba-NS Enterobacterales meningitis with active agents, the clinical efficacy rate of the aminoglycoside-based (including amikacin [*n* = 21, administered as 0.8 g q24h ivgtt], gentamicin [*n* = 4], isepamicin [*n* = 4]) combination therapy group (69.0%, 20/29) was higher than that of the non-aminoglycosides therapy group (38.7%, 12/31, *P* = 0.019). Among the 29 cases in the aminoglycoside-based therapy group, 23 received aminoglycosides by intravenous injection alone, 3 received aminoglycosides by both intravenous and intrathecal/intraventricular routes, and 3 received aminoglycosides by the intrathecal/intraventricular route alone. Carbapenems (administered as meropenem 2 g q8h ivgtt with prolonged infusion for 2–3 h) were the most commonly used antimicrobial agent in aminoglycoside-based combination therapy (75.9%, 22/29), and the clinical efficacy rate of the carbapenem-aminoglycoside-based group (68.2%, 15/22) was higher than that of the carbapenem-based group (without aminoglycosides) (22.2%, 4/18, *P* = 0.004). Moreover, the clinical efficacy rate of the SXT (two tablets, oral, twice a day, with 400 mg of SMX and 80 mg of TMP per tablet)-based therapy group (81.8%, 9/11) was higher than that of the non-SXT therapy group (46.9%, 23/49, *P* = 0.036) (Table 3). The clinical efficacy of regimens with fosfomycin (58.8%, 10/17) vs. regimens without fosfomycin (51.2%, 22/43) showed no significance (*P* = 0.592).

**Table 3:** Treatment regimens and clinical outcomes of carba-NS Enterobacterales and carba-NS *Acinetobacter baumannii* complex meningitis

Antimicrobial therapies	Total, n	Clinical success, n (%)	Clinical failure, n (%)	*P*
Carba-NS Enterobacterales meningitis (*n* = 60)				
Aminoglycoside-based *vs.* non-aminoglycosides				
Aminoglycoside-based therapy	29	20 (69.0)	9 (31.0)	**0.019**
Non-aminoglycosides therapy	31	12 (38.7)	19 (61.3)	
SXT-based *vs.* non-SXT				
SXT-based therapy	11	9 (81.8)	2 (18.2)	**0.036**
Non-SXT therapy	49	23 (46.9)	26 (53.1)	
Fosfomycin-based *vs.* non-fosfomycin				
Fosfomycin-based therapy	17	10 (58.8)	7 (41.2)	0.592
Non-fosfomycin therapy	43	22 (51.2)	21 (48.8)	
Carbapenem-based *vs.* aminoglycoside-based therapy				
Carbapenem-based (without aminoglycosides) therapy	18	4 (22.2)	14 (77.8)	
Aminoglycoside-based (without carbapenems) therapy	7	5 (71.4)	2 (28.6)	**0.007**
Carbapenems + aminoglycoside-based	22	15 (68.2)	7 (31.8)	
Carba-NS *A. baumannii* complex meningitis (*n* = 82)				
Tetracycline-based *vs.* non-tetracyclines				
Tetracycline-based therapy	35	22 (62.9)	13 (37.1)	**0.044**
Non-tetracycline therapy	47	19 (40.4)	28 (59.6)	
Sulbactam-based *vs.* non-sulbactam				
Sulbactam-based therapy	40	21 (52.5)	19 (47.5)	0.659
Non-sulbactam therapy	42	20 (47.6)	22 (52.4)	
Carbapenem-based *vs.* sulbactam-based therapy				
Carbapenem-based (without sulbactam) therapy	29	11 (37.9)	18 (62.1)	
Sulbactam-based (without carbapenems) therapy	23	13 (56.5)	10 (43.5)	0.409
Carbapenems + sulbactam-based therapy	17	8 (47.1)	9 (52.9)	

Additionally, multivariate analysis showed that the use of aminoglycosides was associated with improved efficacy (OR: 3.519; 95% CI: 1.209–10.24; *P* = 0.021) (see Additional file 1). Cox regression showed that the use of aminoglycosides was an independent factor for survival (hazard ratio [HR]: 0.371; 95% CI: 0.144–0.952; *P* = 0.039) (Fig. 4C). However, Cox regression showed that SXT was not significantly associated with survival (HR: 0.028; 95% CI: 0.000–1.610; *P* = 0.084) (see Additional file 2).

### Carba-NS A. *Baumannii* complex meningitis

For the 124 cases of *A. baumannii* complex meningitis, the clinical success rate was not statistically significant between the Carba-NS group (45.1%, 41/91) and Carba-S group (51.5%, 17/33; *P* = 0.524). These patients were commonly afflicted by *A. baumannii* complex pneumonia and bloodstream infections; 51 (56.0%) and 8 (8.8%) cases for the Carba-NS group, respectively, and for the Carba-S group, 16 (48.5%) cases were co-infected with *A. baumannii* complex pneumonia. Among the 91 patients with Carba-NS *A. baumannii* complex meningitis, 82 (90.1%) received therapy involving active agents. All nine patients who received inappropriate therapy without active agents failed to respond to the treatment. The clinical efficacy rates of patients who received monotherapies (41.7%, 5/12) and combination therapies (51.4%, 36/70) showed no statistical significance (*P* = 0.532). Comparing the 2004–2009 group with the 2010–2019 group, the clinical efficacy rates (45.2% [19/42] vs. 47.6% [39/82]) did not show significant differences (*P* = 0.806).

For the 82 cases of Carba-NS *A. baumannii* complex meningitis with active agents, the clinical efficacy rate of the tetracycline-based (including tigecycline [*n* = 21, the first dosage was 100 mg, followed by 50 mg q12h ivgtt], doxycycline [*n* = 12], and minocycline [*n* = 2]) therapy group (62.9%, 22/35) was higher than that of the non-tetracycline therapy group (40.4%, 19/47, *P* = 0.044) (Table 3). Multivariate analysis indicated that tetracyclines were associated with efficacy improvement (OR: 2.494; 95% CI: 1.014–6.132; *P* = 0.047) (see Additional file 3). Cox regression showed that the use of tetracyclines was not an independent factor for survival (HR: 0.930; 95% CI: 0.501–1.726; *P* = 0.817).

The clinical efficacy rates were not statistically significant between the sulbactam-based (administered as cefoperazone 2.0 g plus sulbactam 1.0 g q8h ivgtt) therapy group (52.5%, 21/40) and the non-sulbactam therapy group (47.6%, 20/42; *P* = 0.659). Meanwhile, the clinical efficacy rates of the carbapenem-based group, sulbactam-based group, and carbapenem-sulbactam-based group were 37.9% (11/29), 56.5% (13/23), and 47.1% (8/17), respectively. No statistical significance was observed among these three groups (*P* = 0.409).

### *S. maltophilia* meningitis

Analysis of 16 *S. maltophilia* cases showed that the clinical success rate was 68.8% (11/16) and the in-hospital mortality rate was 12.5% (2/16). Levofloxacin, SXT, and tetracyclines were the most commonly used antibacterial agents. No statistical significance was observed among the different therapy groups.

## Discussion

In this study, 505 Gram-negative bacilli were isolated from 505 patients, 83.8% of whom were post-neurosurgical patients. Two of the most common isolated Gram-negative bacilli were *Acinetobacter* spp. and *Klebsiella* spp., which had meropenem-resistance rates of 50.6% and 42.5%, respectively, and showed a growing carbapenem-resistance trend during this 16-year period. The increasing carbapenem-resistance trends for isolates from the CSF are in accordance with the bacterial resistance trends for isolates collected from all specimens in China. The prevalence of carbapenem-resistant *K. pneumoniae* and CRAB increased from 3 to 25% and from 33 to 77% from 2005 to 2018, respectively, as reported by the China antimicrobial surveillance network (CHINET) [8]. However, the prevalence of carbapenem-resistant *K. pneumoniae* (64%) and CRAB (85%) from the CSF was higher than those from three common specimens (lower respiratory tract, urine, and blood; 19–34% and 42–80%, respectively) [8]. The increasing trends of carbapenem-resistance during the study period may be driven by several factors, including the intensive use of antibacterials.

We analyzed the characteristics and risk factors of Carba-NS Gram-negative bacterial meningitis compared to Carba-S Gram-negative bacterial meningitis. Previous studies have found risk factors contributing to healthcare-associated meningitis, such as CSF shunt, CSF drains, and intrathecal infusion pumps [17]. The current study is the first to reveal the risk factors of Carba-NS Gram-negative bacterial meningitis, including the GCS score, non-CNS infections on admission, application of ICP monitoring, and carbapenem exposure.

In previous studies, the mortality rates of Gram-negative bacterial meningitis have been shown to range from 8.7 to 59.1% [19, 20]. This study indicated that the Carba-NS group showed a poorer outcome and a higher in-hospital mortality rate (18.8%, 35/186) compared to those of the Carba-S group (7.4%, 9/122, *P* = 0.005). Notably, all-cause mortality is related to many factors, including comorbidities and complications. In this study, Carba-NS patients were more likely to have poor healthy conditions, including poor state of consciousness and more non-CNS infections, as indicated in Table 2. These factors may contribute to the observed higher mortality in Carba-NS patients. Consequently, though the all-cause mortality increased in the Carba-NS group, there was no significant difference in clinical success between the two groups.

With the limited availability of active antibacterial agents and the existence of the blood-brain barrier, treatment of Carba-NS Gram-negative bacterial meningitis is challenging and the outcome is unfavorable. Thorough basic life support and nursing care may be beneficial to improve the outcome. Moreover, the use of active antimicrobial agents and the development and accessibility of novel antimicrobial agents are of greater importance.

Regarding the treatment of Carba-NS Enterobacterales meningitis, our results revealed that the clinical efficacy rate of the aminoglycoside-based group was higher than that of the non-aminoglycoside-based group, and aminoglycosides were an independent factor for survival. The amikacin susceptible rate for carbapenemase-producing Enterobacterales has been shown to be 75.2–93.5% in the Asian-Pacific region [21]. In our set, the susceptible rates of *Klebsiella* spp. (*n* = 106) and other Enterobacterales (*n* = 39) to amikacin were 49.1% and 76.9%, respectively. Aminoglycosides such as amikacin and gentamicin are recommended either for intrathecal/intraventricular [17, 22, 23] or for intravenous use along with systemically administered antibiotics (such as carbapenems) [24–26] to treat meningitis caused by Gram-negative bacteria, especially multi-drug resistant or carbapenem-resistant bacteria. Although the CSF penetration of aminoglycosides is considered to be particularly poor, the blood-CSF/blood-brain barrier becomes leaky in the context of meningitis inflammation, leading to an increase in drug concentrations within CSF. This increased permeability makes it possible for intravenously administered drugs to exert effects [23]. Moreover, aminoglycosides are usually applied as an addition to β-lactam antibiotics, which shows a synergistic activity and forms a classic combination therapy for critically ill patients with Gram-negative infection. In this study, 22 of the 29 patients who received aminoglycosides were administered β-lactam antibiotics. Though doubted by some studies [27], this β-lactam-aminoglycosides combination is supported by in vitro and in vivo experimental data [28, 29] and clinical studies [30–33].

SXT may be useful for treating CRAB and methicillin-resistant *Staphylococcus aureus* infection [34]. The penetration of SXT into the CSF in both the absence and presence of meningeal inflammation is higher than that of β-lactam antibiotics and aminoglycosides [23]. In this study, the susceptible rates of *Klebsiella* spp. and other Enterobacterales to SXT were 56.6% and 46.2%, respectively, in accordance with the findings of previous reports [8]. Efficacy analysis demonstrated the superiority of SXT-based therapy for treating Carba-NS Enterobacterales meningitis, although Cox regression found no statistically significant difference from non-SXT therapy.

For the treatment of Carba-NS *A. baumannii* meningitis, our findings revealed that the clinical efficacy of the tetracycline-based therapy group was higher than that of the non-tetracycline therapy group, presumably due to the relatively high susceptibility of *A. baumannii* spp. to minocycline and tigecycline (48.8% and 52.4%, respectively). Though the CSF penetration of tetracyclines is generally regarded as limited, guidelines recommend doxycycline for meningitis caused by microorganisms such as *Mycoplasma* and *Leptospira* [25]. Case reports and in vitro studies indicate that tetracyclines, as monotherapy and in combination, represent an alternative strategy in the salvage treatment of multidrug-resistant *Acinetobacter* spp. meningitis [35–39]. Additionally, patients with Carba-NS *A. baumannii* complex meningitis were commonly afflicted by *A. baumannii* complex pneumonia and bloodstream infections, accounting for 64.8% (59/91) in this study. Tetracyclines are also helpful in treating other *A. baumannii* complex infections besides meningitis.

Polymyxins are recommended for the treatment of CRAB infections, with a previous study showing that a long course of colistin therapy may have better clinical and microbiological outcomes compared to a short course in critical patients [40]. However, colistin is not an ideal antibiotic for the treatment of meningitis because of its poor blood-brain barrier penetration; indeed, a survey showed that 20% (11/56) of countries have no access to colistin [41]. In China, polymyxins were not available for clinical use before 2018, and they were costly after 2018, and thus, they were prescribed only in combination for 17 patients with Carba-NS Enterobacterales and *A. baumannii* meningitis. New antibacterials such as ceftazidime-avibactam may have satisfactory efficacy for the treatment of CRKP meningitis [42]; however, patient access to new antibacterials is limited not only in low- and middle-income countries but also in some high-income countries [43]. Sulbactam-durlobactam [44] exhibited promising prospects in the treatment for CRAB infections and was approved in 2023. Further studies are needed to establish whether this new drug could be used for the treatment of CRAB meningitis. In this study, we show that combination therapies involving old antibiotics such as aminoglycosides, trimethoprim-sulfamethoxazole, and tetracyclines may improve patients’ outcome for the treatment of Carba-NS *K. pneumoniae* or *A. baumannii* meningitis. These findings have implications for clinical treatment strategies globally, especially in areas with limited healthcare resources, and provide valuable insights for decision-making.

## Conclusions

Our results revealed the characteristics of Carba-NS Gram-negative bacterial meningitis, which was found to be associated with higher in-hospital mortality rates. Combination therapies involving old antibiotics such as aminoglycosides, trimethoprim-sulfamethoxazole, and tetracyclines may improve patients’ outcome, which is informative for the management of Carba-NS bacterial meningitis when resource is constrained.

### Supplementary Information

**Supplementary Material 1.**

**Supplementary Material 2.**

**Supplementary Material 3.**

## Data Availability

The datasets used in this study are available from the corresponding author on reasonable request.

## Abbreviations

CNS: Central nervous system
CHINET: China Antimicrobial Resistance Surveillance Network
CSF: Cerebrospinal fluid
CRE: Carbapenem-resistant Enterobacterales
SXT: Trimethoprim-sulfamethoxazole
Carba-NS: Carbapenem-non-susceptible
Carba-S: Carbapenem-susceptible
CDC/NHSN: Center for Disease Control and Prevention and National Healthcare Safety Network
CLSI: Clinical and Laboratory Standards Institute
MICs: Minimal inhibitory concentrations
GCS: Glasgow Coma Scale
GOS: Glasgow Outcome Scale
ICU: Intensive care unit
WBC: White blood cell
ICP: Intra-cranial pressure
PPI: Proton pump inhibitor
VP: Ventriculoperitoneal
OR: Odds ratio
