# Supplementary material for: Clinical characteristics and antimicrobial therapy of healthcare-associated carbapenem-non-susceptible gram-negative bacterial meningitis: a 16-year retrospective cohort study
Source: BMC Infect Dis. 2024 Apr 2;24:368. doi: 10.1186/s12879-024-09237-9 (PMC10985894; doi:10.1186/s12879-024-09237-9)
Supplement: Supplementary file 1 — Supplementary Material 1. [file 12879_2024_9237_MOESM1_ESM.docx]

Logistic regression analysis of treatment options associated with efficacy of Carba-NS Enterobacteriales meningitis

|  | Univariate Analysis | | Multivariate Analysis | |
| --- | --- | --- | --- | --- |
|  | OR (95% CI) | *P* | OR (95% CI) | *P* |
| Carbapenems | 0.429 (0.142-1.293) | 0.133 |  |  |
| Aminoglycosides | 3.519 (1.209-10.240) | **0.021** | 3.519 (1.209-10.240) | **0.021** |
| Tigecycline | 0.600 (0.197-1.825) | 0.368 |  |  |
| Polymyxins | 0.379 (0.085-1.687) | 0.203 |  |  |
| Fosfomycin | 1.364 (0.438-4.247) | 0.593 |  |  |
| Trimethoprim-sulfamethoxazole | 5.087 (0.995-26.006) | 0.051 |  |  |
