# Supplementary material for: Clinical characteristics and antimicrobial therapy of healthcare-associated carbapenem-non-susceptible gram-negative bacterial meningitis: a 16-year retrospective cohort study
Source: BMC Infect Dis. 2024 Apr 2;24:368. doi: 10.1186/s12879-024-09237-9 (PMC10985894; doi:10.1186/s12879-024-09237-9)
Supplement: Supplementary file 2 — Supplementary Material 2. [file 12879_2024_9237_MOESM2_ESM.docx]

Cox survival analysis of factors associated with in-hospital death of Carba-NS Enterobacteriales meningitis patients

|  |  | Univariate Analysis | | Multivariate Analysis | |
| --- | --- | --- | --- | --- | --- |
|  |  | OR (95% CI) | *P* | OR (95% CI) | *P* |
| Male sex | | 0.619 (0.252-1.520) | 0.296 |  |  |
| Age | | 1.004 (0.975-1.033) | 0.789 |  |  |
| Diabetes | | 3.150 (0.873-11.36) | 0.080 |  |  |
| Hypertension | | 1.882 (0.789-4.492) | 0.154 |  |  |
| Cerebrovascular disease | | 0.753 (0.205-2.767) | 0.669 |  |  |
| Traumatic brain injury | | 0.445 (0.112-1.763) | 0.249 |  |  |
| Intracranial tumor | | 0.282 (0.055-1.443) | 0.129 |  |  |
| With non-CNS infection on admission | | 2.292 (0.972-5.405) | 0.058 |  |  |
| Hospitalization in the previous month before admission | | 1.379 (0.566-3.362) | 0.479 |  |  |
| Ward | |  |  |  |  |
|  | Neurosurgery | 1.843 (0.240-14.182) | 0.557 |  |  |
|  | ICU | 3.723 (0.432-32.072) | 0.232 |  |  |
|  | Infectious disease | 3.325 (0.344-32.092) | 0.299 |  |  |
| Organ failure | | 1.828 (0.804-4.159) | 0.150 |  |  |
| Mechanical ventilation | | 3.137 (1.161-8.476) | **0.024** |  |  |
| External ventricular drainage | | 1.203 (0.525-2.757) | 0.662 |  |  |
| Ventriculoperitoneal shunt | | 0.597 (0.174-2.053) | 0.413 |  |  |
| Intra-cranial pressure monitor | | 1.449 (0.590-3.558) | 0.419 |  |  |
| Ommaya | | 1.376 (0.587-3.225) | 0.463 |  |  |
| Continuous lumbar drainage | | 0.619 (0.267-1.437) | 0.264 |  |  |
| Glucocorticoids | | 1.040 (0.454-2.380) | 0.927 |  |  |
| Proton pump inhibitor | | 0.877 (0.321-2.399) | 0.798 |  |  |
| Hypoalbuminemia | | 0.872 (0.359-2.116) | 0.762 |  |  |
| Hydrocephalus | | 0.638 (0.267-1.525) | 0.312 |  |  |
| CSF leakage | | 0.629 (0.184-2.155) | 0.461 |  |  |
| Surgical wound infection | | 0.575 (0.191-1.735) | 0.326 |  |  |
| Co-infection with Gram-positive bacteria or MTB in CNS | | 0.787 (0.324-1.908) | 0.595 |  |  |
| Carbapenems | | 1.645 (0.641-4.218) | 0.300 |  |  |
| Aminoglycosides | | 0.379 (0.150-0.962) | **0.041** | 0.371 (0.144-0.952) | **0.039** |
| Tigecycline | | 1.588 (0.656-3.843) | 0.305 |  |  |
| Polymyxins | | 1.672 (0.611-4.577) | 0.317 |  |  |
| Fosfomycin | | 0.910 (0.354-2.339) | 0.845 |  |  |
| Trimethoprim-sulfamethoxazole | | 0.028 (0.000-1.610) | 0.084 |  |  |

MTB, mycobacterium tuberculosis.
