# Supplementary material for: Clinical characteristics and antimicrobial therapy of healthcare-associated carbapenem-non-susceptible gram-negative bacterial meningitis: a 16-year retrospective cohort study
Source: BMC Infect Dis. 2024 Apr 2;24:368. doi: 10.1186/s12879-024-09237-9 (PMC10985894; doi:10.1186/s12879-024-09237-9)
Supplement: Supplementary file 3 — Supplementary Material 3. [file 12879_2024_9237_MOESM3_ESM.docx]

Logistic regression analysis of treatment options associated with efficacy of Carba-NS *Acinetobacter baumannii* complex meningitis

|  | Univariate Analysis | | Multivariate Analysis | |
| --- | --- | --- | --- | --- |
|  | OR (95% CI) | *P* | OR (95% CI) | *P* |
| Carbapenems | 0.512 (0.214-1.223) | 0.132 |  |  |
| Sulbactam (and its fixed-dose combination) | 1.326 (0.562-3.130) | 0.519 |  |  |
| Aminoglycosides | 0.663 (0.277-1.589) | 0.357 |  |  |
| Tetracyclines (tigecycline, doxycycline and minocycline) | 2.494 (1.014-6.132) | **0.047** | 2.494 (1.014-6.132) | **0.047** |
| Polymyxins | 1.054 (0.246-4.525) | 0.944 |  |  |
| Fosfomycin | 0.800 (0.281-2.280) | 0.676 |  |  |
| Trimethoprim-sulfamethoxazole | 1.618 (0.256-10.219) | 0.609 |  |  |
